# Lower Serum HBV RNA Level is Associated with Liver Cirrhosis in Patients Treated with Nucleos(t)ide Analogs: A Population-Based Cross-Sectional Study

**DOI:** 10.5152/tjg.2025.24648

**Published:** 2025-03-18

**Authors:** Juanli Wu, Yushuang Zhang, Han Gao, Yiheng Zhang, Tao Li, Lei Wang, Yundong Qu

**Affiliations:** The Second Hospital of Shandong University, Jinan, China

**Keywords:** Cirrhosis, HBsAg, HBV DNA, HBV RNA, Hepatitis B virus, Nucleos(t)ide analogs

## Abstract

**Background/Aims:**

There is evidence suggesting an association between hepatitis B virus (HBV) RNA and hepatic fibrosis in treatment-naïve chronic hepatitis B (CHB) patients. However, few studies have delved into the relevance between HBV RNA and HBV-related cirrhosis. The purpose of this article is to elucidate the connection between HBV RNA and cirrhosis in patients undergoing nucleos(t)ide analogs (NAs).

**Materials and Methods:**

This study included 381 patients. Logistic regression was employed to investigate the variables linked to the development of cirrhosis. Propensity score matching was used to correct for confounders.

**Results:**

In this cross-sectional study, multivariable logistic regression showed HBV RNA detectability was associated with cirrhosis. Multivariate regression suggests that the variables associated with cirrhosis were: older age, HBV RNA above the lower limit of quantification, shorter duration of treatment, higher level of serum bilirubin, and treatment strategy. Among patients with quantifiable HBV RNA (n = 242), cirrhotic patients exhibit lower median serum HBV RNA titers compared to those with CHB patients. Furthermore, qHBsAg ≤ 3.3 lg IU/mL and qHBV RNA ≤ 3.7 lg copies/mL may be linked to cirrhosis.

**Conclusion:**

Nucleos(t)ide analogs treatment may result in varying degrees of decrease in HBV RNA levels. Lower levels of HBV RNA may be linked to the development of cirrhosis.

## Main Points

- Hepatitis B virus (HBV) RNA has an excellent ability to reflect the replication activity of the virus and to assess the disease process.
- Hepatitis B-associated cirrhosis remains a major threat to human health, and its early noninvasive diagnosis needs to be continuously optimized.
- Lower levels of HBV RNA have been observed to be associated with cirrhosis in patients on long-term nucleos(t)ide analog therapy.

## Introduction

Chronic hepatitis B (CHB) is a worldwide public health issue, affecting approximately 296 million individuals worldwide.^1^ Patients with CHB are more likely to develop cirrhosis and even hepatocellular carcinoma (HCC).

While nucleos(t)ide analogs (NAs) and pegylated interferon are currently classified as first-line therapies for CHB,^2^ a complete cure is still unachievable due to the presence of covalent closed-circle DNA (cccDNA). The direct detection of intrahepatic cccDNA requires an invasive liver biopsy, while traditional indices such as hepatitis B virus (HBV) DNA, hepatitis B surface antigen (HBsAg), hepatitis B e antigen (HBeAg), etc. are used to indirectly reflect the replicative activity of the virus.

For the past few years, HBV RNA has emerged as a novel marker for assessing viral replicative activity. It has been proven manifestly advantageous in predicting the effectiveness of NAs, strategizing the timing of discontinuation, and identifying potential instances of relapse after discontinuation,^3^^-^^7^ and elevated HBV RNA levels increase the risk of liver cancer.^8^ Although previous studies have explored the potential connection between serum HBV RNA and liver fibrosis in treatment-naïve CHB patients,^9^ few study has delved into the correlation between serum HBV RNA and cirrhosis in NAs-treated patients. The schematic diagram of the article was to evaluate the distribution of HBV RNA in patients with CHB and cirrhosis, and to explore its possible association with the development of cirrhosis.

## Materials and Methods

### Enrolled Patients

This cross-sectional study gathered data on registered patients who visited the Department of Hepatology’s outpatient and inpatient clinics at the Second Hospital of Shandong University from June 2006 to September 2023. The data were obtained from the HIS electronic medical record system and included parameters such as HBV DNA, HBV RNA, and routine biochemical tests.

**Figure 1.**
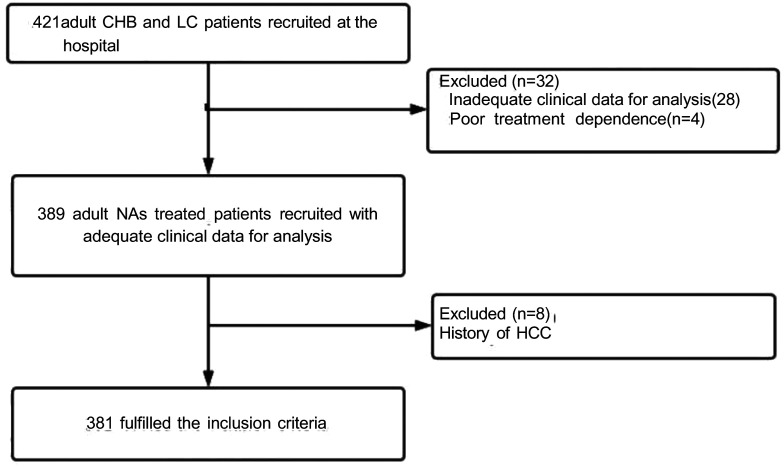
Flow diagram of participant selection. CHB, chronic hepatitis B; HCC, hepatocellular carcinoma; LC, liver cirrhosis; NAs, nucleos(t)ide analogs.

Patients who were eligible for inclusion were (1) greater than 18 years old, (2) previously HBsAg-positive for over 6 months, and (3) undergoing TFV undergoing TFV [including TDF (tenofovir disoproxil fumarate) and TAF (tenofovir alafenamide fumarate)] or entecavir (ETV) antiviral therapy. Exclusion criteria: (1) poor treatment adherence, (2) co-infection with hepatitis C or D virus or human immunodeficiency virus, combined with alcoholic hepatic disease, nonalcoholic fatty liver disease, autoimmune liver disease, or (3) occurrence of HCC or acute or chronic hepatic failure. The flowchart of the inclusion of cases is shown in Figure 1. To facilitate analysis, patients were categorized into CHB and cirrhosis groups based on the clinical practice guidelines of the American Association for the Study of Liver Diseases.^2^ Informed consent was obtained from the patients and the study was ethically reviewed by the Second Hospital of Shandong University (approval no: KYLL-2023-431, date: November 20, 2023).

### Laboratory Measurements

Hepatitis B surface antigen and HBeAg were quantified by the i2000 Chemiluminescent Immunoassay analyzer (Abbott, USA) and the Abbott reagents.

### Detection of Serum Hepatitis B Virus DNA

Circulating HBV DNA was measured by real-time polymerase chain reaction assay using Roche COBAS TaqMan (Basel, Switzerland), with the lower limit of quantification [LLQ] 20 IU/mL.

### Measurement of Serum Hepatitis B Virus RNA

Serum HBV RNA was measured by specific RNA target capture and simultaneous amplification and testing (Rendu Biotechnology, Shanghai, China), which avoids interference from mixed DNA in the RNA extraction process. The LLQ is 100 copies/mL, and the specific procedure for HBV RNA testing was referenced from published studies.^10^

### Statistical Analysis

Continuous variables were expressed as mean ± SD or median [interquartile range (IQR)]. Categorical data were expressed as counts and/or proportions. Log-transformed expressions of HBsAg, HBV DNA, and HBV RNA were analyzed to compare continuous variables between the 2 groups using the Student’s *t*-test or Mann–Whitney *U*-test. Logistic regression was utilized for univariate and multivariate analyses to identify variables linked to the development of cirrhosis and their relationship with the serum level of HBV RNA. Propensity score matching (PSM) was used to balance the confounding variables (matching ratio 1 : 1; caliper value = 0.02; counterbalancing variables included age, gender, treatment regimen, treatment duration, ALT, and TB; 58 pairs of patients were eventually matched). The R Statistical Software Version 4.2.2 (http://www.R-project.org, The R Foundation, Beijing, China) and Free Statistics analysis platform (Version 1.8, Beijing, China) were used in data analysis. A bilateral test with a *P*-value of less than .05 was considered statistically significant.

## Results

### Baseline Characteristics

The basic clinical information of 381 patients is described in Table 1, with an average age of 48.2 years and 265 (69.6%) males, more males than females. All patients received first-line NAs therapy, with 170 (44.6%) receiving ETV and 211 (55.4%) receiving TFV. The median duration of antiviral therapy for all patients was 55 months.

For the purpose of analysis, classification of patients into CHB and cirrhosis groups based on diagnosis. Cirrhotic patients exhibited higher levels of biochemical parameters such as AST, TB, etc. Serum HBsAg titers were higher in patients with CHB than in those with cirrhosis; however, the 2 groups did not show a statistically significant difference in the duration of NAs treatment or in the detection rates of HBV DNA and HBV RNA.

### Factors Associated with Liver Cirrhosis

Indicators linked to cirrhosis were identified through univariate and multivariate logistic regression analysis (Table 2). Univariate logistic regression analyses have shown that older age, ETV treatment, low qHBsAg levels, higher titers of serum bilirubin and ALT, and HBeAg positive are all associated indicators of cirrhosis. Multivariate logistic regression showed that factors associated with cirrhosis were older age (OR 2.01, *P* = .008), HBV RNA above the lower limit of detection (LLQ) (OR 1.08, *P <* .001), shorter duration of treatment, higher titers of serum bilirubin (OR 2.35, *P* = .002), ETV treatment. Interestingly, lower qHBsAg levels were associated with cirrhosis development (OR 1.08, *P* < .001) and HBV DNA positivity was not connected with the cirrhosis.

### Virologic Factors Associated with the Development of Cirrhosis

**Figure 2.**
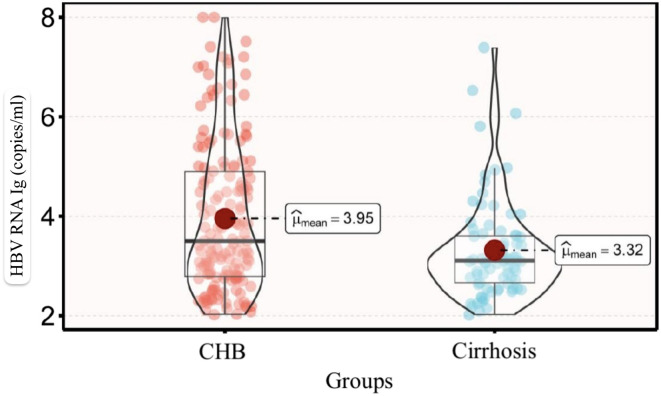
Comparison of circulating qHBV RNA in CHB and cirrhosis patients. Abbreviation: CHB, chronic hepatitis B.

Two hundred forty-two patients with quantifiable HBV RNA were further analyzed, and their clinical characteristics are described in Table 3. In patients with cirrhosis, qHBV RNA titers were lower than in those with CHB. Figure 2 provides a visual representation of the qHBV RNA distribution in the two groups. To describe the relationship between the viral replication indicator and cirrhosis, PSM was utilized to equalize confounders, including gender, age, treatment duration, and treatment regimen. Multivariate logistic regression revealed that qHBsAg ≤ 3.3 lg IU/mL and qHBV RNA ≤ 3.7 lg copies/mL were closely linked to the progression of cirrhosis (Table 4).

**Figure 3.**
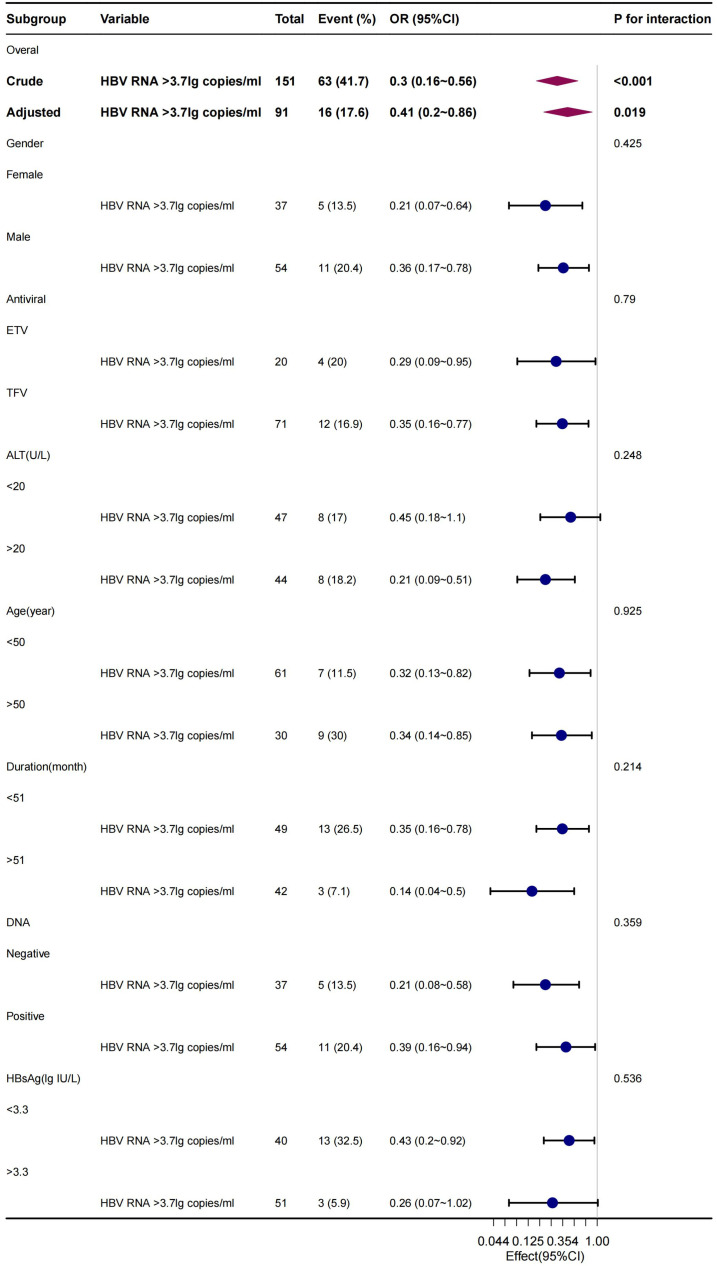
Multivariate logistic regression subgroup analysis of the pertinence between qHBV RNA and cirrhosis across gender, age, treatment regimen, ALT, HBV DNA, and qHBsAg.

**Figure 4.**
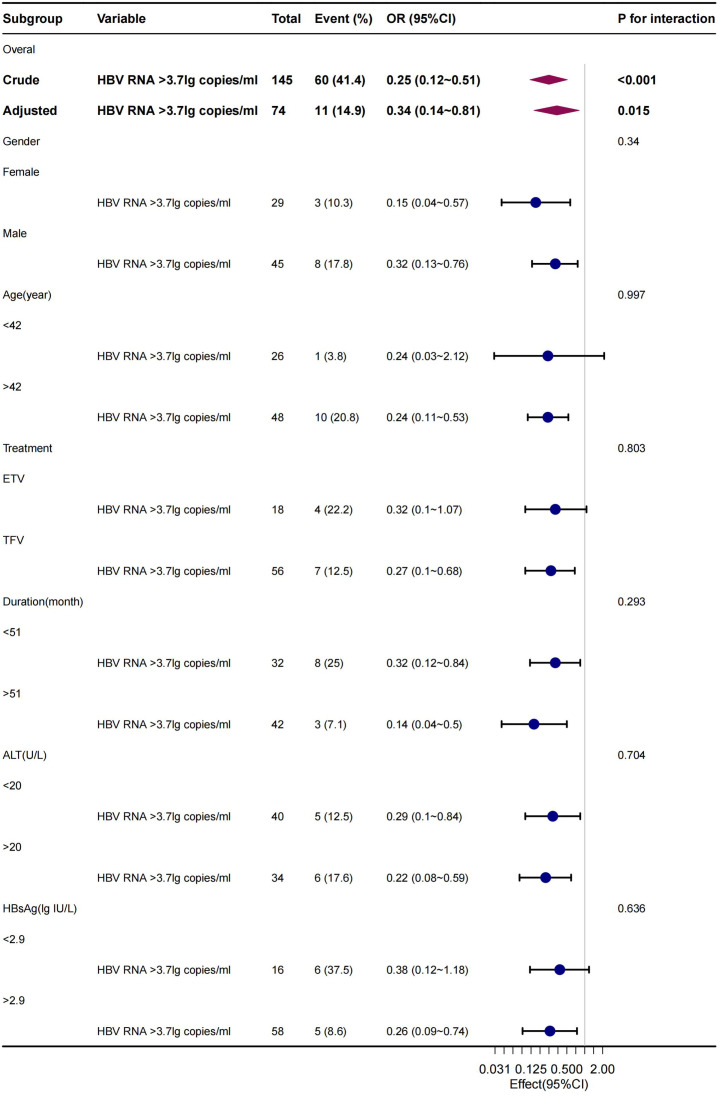
Multivariate logistic regression subgroup analysis of the pertinence between qHBV RNA and cirrhosis in patients treated for more than 12 months across gender, age, treatment regimen, ALT, and qHBsAg.

The stability and applicability of the conclusions were further examined through subgroup analyses on populations of different sexes, ages, and treatment regimens. The analyses revealed that lower levels of qHBV RNA were significantly linked to cirrhosis (Figure 3). This association was consistently observed in the subgroup of individuals treated with NAs for more than 12 months (Figure 4), affirming the robustness of the findings.

## Discussion

This study examines the distribution of HBV RNA in CHB patients and its relationship with cirrhosis. After long-term NAs therapy (with a mean duration of 55 months), it was discovered that the detection rate of HBV RNA (63.5%) was significantly higher than that of HBV DNA (33.6%) (Supplementary Table 1). It was further quantified through the application of multivariate logistic regression analysis, which delineated a distinctive association between detectable HBV RNA and cirrhosis. Our subsequent investigation, which focused on the 242 patients with HBV RNA above the LLQ, indicates that the serum qHBV RNA levels were modestly reduced in cirrhotic patients compared with patients diagnosed with CHB. This, in turn, enriches the understanding of the disease process and guides the approach towards personalized patient care.

As a framework for viral replication, cccDNA is processed into RNA fragments of varying dimensions before being released into the cytoplasm. Therein, these fragments transform into capsid-enclosed pre-genomic RNA (pgRNA). Despite the transformation of pgRNA into relaxed circular DNA being thwarted by NAs, these NAs do not explicitly deter the synthesis of HBV RNA.^11^^-13^ Previous investigations into the kinetics of HBV RNA substantiate these findings.^14^ During the treatment, the decrement in HBV RNA was epitomized in 2 distinct phases. It is in the latter phase that, while HBV DNA eluded detection, there was a slow yet continued decline in HBV RNA. The present research also showed that among patients undergoing NA treatment, HBV RNA had a higher detection rate than HBV DNA.

This study focused on investigating the relationship between virological indicators and cirrhosis. Using PSM, potential confounders such as age, sex, treatment duration, and antiviral regimen were adjusted for. Multivariate logistic regression analysis revealed that lower level of qHBsAg (≤3.3 lg IU/mL) had a significant correlation with the stage of cirrhosis. Previous research also supports these findings, indicating that lower qHBsAg levels are associated with increased severity of hepatic fibrosis and cirrhosis in HBeAg-positive treatment-naive patients.^15^ Nevertheless, there is a discrepancy in the findings related to HBeAg-negative patients. Prevailing studies propose the lack of a correlation in this category; however, our results show otherwise. The primary distinction could be attributed to our study’s inclusion of treated patients, with a median treatment duration of more than 4 years. Furthermore, research conducted on primary cirrhosis patients revealed an inverse association between qHBsAg and the collagen proportionate area (CPA) in liver tissue.^16^ Importantly, CPA levels are a better indicator of the extent of cirrhosis. This relationship provides additional understanding of how qHBsAg influences the progression of cirrhosis.

The association between a lower level of qHBV RNA (≤3.7 lg copies/mL) and cirrhosis was observed in our study. This finding aligns with prior research, which indicates an inverse correlation between serum qHBV RNA and Ishak fibrosis score in HBeAg-positive patients. This suggests that significant reductions in HBV RNA at the sixth month of therapy can predict subsequent liver fibrosis regression.^17^ It is important to note, however, that the sensitivity of this conclusion is only 53.8%. The study has limitations as it exclusively focused on HBeAg-positive patients with abnormal aminotransferases and high serum levels of qHBV DNA, thus limiting the generalizability of its findings. Additionally, other researchers also propose a relationship between lower qHBV DNA and the fibrosis progression.^18^ This is complicated by the fact that some patients treated with NAs experience rapid HBV DNA clearance but still progress towards cirrhosis, causing uncertainty about the association between low levels of qHBV DNA and cirrhosis development. Furthermore, another independent study suggests that serum qHBV RNA is a superior predictor of hepatic fibrosis severity in naive CHB patients compared to APRI and FIB-4.^9^ However, this study did not include post-treatment patients in its cohort, potentially limiting the generalizability of its findings. Considering the impact of NAs efficacy and baseline HBV DNA titer on study outcomes, the characteristics of patients undergoing NAs for more than 12 months were analyzed and found that only 4.1% (9/219) of patients had HBV DNA higher than the LLQ. The subgroup results showed that, regardless of variations in age, treatment regimen, ALT levels, and other potential confounders, lower levels of HBV RNA were consistently associated with cirrhosis. This finding is stable and applicable.

The multivariate logistic regression analysis indicated several significant indicators associated with cirrhosis, including older age (>50 years), shorter duration of treatment (<46 months), high serum bilirubin levels, and ETV treatment. Notably, older age is a key component of the FIB-4 index, and individuals with advanced age are at a heightened risk of developing cirrhosis, with an increased susceptibility to progression to HCC, which was also demonstrated by another study.^19^ Moreover, elevated serum bilirubin levels are known to escalate the risk of liver fibrosis progression. Additionally, high levels of direct bilirubin (DB) have been found to lead to a reduction in mucosal-associated invariant T cells, which may play a pivotal role in antiviral defense.^20^^,^^21^ Consequently, insufficient therapy to suppress viral replication, coupled with chronic liver inflammation and an impaired immune response, can contribute to the development of liver fibrosis.^2^ Notably, among patients undergoing long-term treatment, those receiving ETV exhibited a higher incidence of cirrhosis and were more prone to the development of HCC.^22^

The present study has significant clinical implications for the early noninvasive assessment and recognition of cirrhosis. Nonetheless, it is crucial to emphasize that liver fibrosis and cirrhosis can still develop despite decreased levels of HBV RNA. The precise mechanism that connects low serum levels of qHBV RNA to the onset and progression of liver cirrhosis remains elusive to date.

There are several limitations. Firstly, due to the assay methodology implemented, it is imperative to mention that the LLQ was set at 100 copies/mL. Consequently, we were unable to establish a definitive correlation between qHBV RNA levels below this threshold and the progression of cirrhosis. This study established only an association between levels of quantitative HBV RNA and cirrhosis. In addition, establishing causality in this investigation is inherently challenging, primarily because the nature of this inquiry was cross-sectional. This served to establish only an association between quantitative HBV RNA levels and cirrhosis but stopped short of establishing a clear-cut causal relationship. To address this issue, the improvement of detection levels and in-depth research on the transcription and mechanism of action of HBV RNA are necessary.

## Conclusion

After long-term antiviral treatment, patients with HBV infection may experience a reduction in HBV RNA levels to varying degrees, but lower levels of HBV RNA may be associated with the development of cirrhosis.

## Supplementary Materials

Supplementary Material

## Figures and Tables

**Table 1.** Baseline Characteristics

	Total (n = 381)	CHB (n = 259)	Cirrhosis (n = 122)	*P*
Male, n (%)	265 (69.6)	176 (68.0)	89 (73.0)	.323
Age (years)	48.2 ± 11.0	46.2 ± 10.8	52.5 ± 10.4	**<**.**001**
Treatment (n, %)				**<**.**001**
ETV	170 (44.6)	100 (38.6)	70 (57.4)	
TFV	211 (55.4)	159 (61.4)	52 (42.6)	
Follow-up duration (month)	55.0 (31.0, 76.0)	57.0 (33.0, 76.0)	52.5 (30.2, 75.0)	.782
ALT (U/L)	21 (15, 30)	20 (15, 30)	22 (17, 30)	.109
AST (U/L)	21 (17, 25)	19 (16, 24)	23 (20, 28)	**<**.**001**
TB (µmoL/L)	14.2 (11.1, 20.8)	13.6 (10.5, 17.7)	17.4 (12.6, 26.8)	**<**.**001**
DB (µmoL/L)	4.8 (3.8,7.1)	4.5 (3.6,6.0)	6.2 (4.3, 9.4)	**<**.**001**
qHBsAg (lg IU/mL)	3.1 (2.3, 3.5)	3.2 (2.6, 3.7)	2.6 (1.9, 3.1)	**<**.**001**
HBeAg positive n (%)	142 (37.3)	115 (44.4)	27 (22.1)	**<**.**001**
Positive (n, %)				
HBV DNA	21 (5.5)	16 (6.2)	5 (4.1)	.688
HBV RNA	242 (63.5)	163 (62.9)	79 (64.8)	.731

Continuous variables were expressed as mean ± SD or median [interquartile range (IQR)].

ALT, alanine transaminase; AST, aspartate aminotransferase; CHB, chronic hepatitis B; DB, direct bilirubin; ETV, entecavir; HBeAg, hepatitis B e antigen; HBsAg, hepatitis B surface antigen; TB, total bilirubin; TFV, tenofovir; Positive: qHBV DNA ＞20 IU/mL, qHBV RNA＞100 copies/mL.

**Table 2.** Univariate and Multivariate Logistic Analysis of Factors Associated with Cirrhosis

Factors	Univariate Analysis			Multivariate Analysis		
	OR	95% CI	*P*	OR	95% CI	*P*
Age, year	2.90	(1.86, 4.52)	**<**.**001**	1.9	(1.13~3.21)	**.016**
Male	1.27	(0.79~2.05)	.323	0.9	(0.5~1.63)	.723
TFV	0.47	(0.30, 0.72)	**.001**	0.44	(0.26~0.77)	**.004**
Duration (month)	0.75	(0.49~1.17)	.205	0.36	(0.2~0.63)	**<**.**001**
ALT (U/L)	1.81	(1.15~2.84)	**.01**	1.62	(0.95~2.76)	.075
TB (µmoL/L)	3.32	(2.07~5.32)	**<**.**001**	2.44	(1.41~4.22)	**.001**
HBeAg	.036	(0.22, 0.58)	**<**.**001**	0.44	(0.23~0.83)	**.012**
qHBsAg (lg IU/mL)	0.18	(0.1~0.33)	**<**.**001**	0.17	(0.09~0.36)	**<**.**001**
HBV RNA	1.08	(0.69, 1.70)	.731	2.38	(1.32~4.29)	**.004**
HBV DNA	0.65	(0.23~1.81)	.41	1.73	(0.51~5.89)	.383

ALT, alanine transaminase; ETV, entecavir, HBeAg, hepatitis B e antigen; qHBsAg, quantifiable hepatitis B surface antigen; TB, total bilirubin; TFV, tenofovir, propofol tenofovir.

**Table 3.** Characteristics of the Patients with Quantifiable HBV RNA

	Total (n = 242)	CHB (n = 163)	Cirrhosis (n = 79)	*P*
Male n (%)	158(65.3)	104(63.8)	54(68.4)	.486
Age (years)	47.8 ± 11.2	45.3 ± 11.0	52.9 ± 10.0	**<**.**001**
Treatment (n, %)				**.02**
ETV	100 (41.3)	59 (36.2)	25 (31.6)	
TFV	142 (58.7)	104 (63.8)	38 (48.1)	
Duration (month)	54.0 (28.2, 76.0)	60.0 (29.5, 77.0)	45.0 (26.0, 69.0)	.094
ALT (U/L)	21 (15,30)	20.0 (14.0, 29.0)	22.0 (16.0, 30.0)	.206
AST (U/L)	21.0 (17.0, 25.0)	19.0 (16.5, 24.0)	22.0 (19.0, 27.0)	**<**.**001**
TB (µmoL/L)	14.0 (10.6, 20.2)	13.3 (10.1, 17.1)	17.5 (12.2, 24.0)	**<**.**001**
DB (µmoL/L)	4.7 (3.6, 6.7)	4.4 (3.4, 5.7)	5.7 (4.2, 8.8)	**<**.**001**
qHBsAg (lg IU/mL)	3.2 (2.7, 3.7)	3.4 (3.0, 3.8)	2.8 (2.3, 3.2)	**<**.**001**
Positive n (%)				
HBeAg	134 (55.4)	108 (66.3)	26 (32.9)	**<**.**001**
HBV DNA	21 (8.7)	16 (9.8)	5 (6.3)	.816
qHBV RNA (lg copies/mL)	3.3 (2.8, 4.5)	3.5 (2.8, 4.9)	3.1 (2.7, 3.6)	**.005**

Continuous variables were expressed as mean ± SD or median [interquartile range (IQR)].

ALT, alanine transaminase; AST, aspartate aminotransferase; DB, direct bilirubin; ETV, entecavir; HBeAg, hepatitis B e antigen; qHBsAg, quantifiable hepatitis B surface antigen; qHBV RNA, quantifiable HBV RNA; TB, total bilirubin; TFV, tenofovir.

**Table 4.** Multivariate Logistic Analyses of Virological Variables Associated with Cirrhosis after PSM in Patients with Quantifiable HBV RNA

Variables	Multivariate Analysis		
	OR	95%CI	*P*
HBeAg	0.48	(0.16~1.44)	.188
HBV DNA (lg IU/mL)	1.21	(0.28~5.27)	.799
qHBsAg (lg IU/mL)	0.26	(0.1~0.67)	**.005**
qHBV RNA (lg copies/mL)	0.32	(0.1~1)	**<**.**001**

HBeAg, hepatitis B e antigen; HBsAg, hepatitis B surface antigen.

## Data Availability

The data that support the findings of this study are available on request from the corresponding author.
