# Supplementary material for: Lower Serum HBV RNA Level is Associated with Liver Cirrhosis in Patients Treated with Nucleos(t)ide Analogs: A Population-Based Cross-Sectional Study
Source: Turk J Gastroenterol. 2025 Mar 18;36(7):442–9. doi: 10.5152/tjg.2025.24648 (PMC12257797; doi:10.5152/tjg.2025.24648)
Supplement: Supplementary Material [file supplementary_material.pdf]

Supplementary Table 1. Comparison of HBV DNA and HBV RNA detection rates

	Total (n=381)	P value	Cirrhosis (n=122)	P value
Detactable (n,%)		.000		.000
HBV DNA	21(5.5)		5 (4.1)	
HBV RNA	242(63.5)		79(64.8)	
